# Efficient Genome Engineering of *Toxoplasma gondii* Using CRISPR/Cas9

**DOI:** 10.1371/journal.pone.0100450

**Published:** 2014-06-27

**Authors:** Saima M. Sidik, Caroline G. Hackett, Fanny Tran, Nicholas J. Westwood, Sebastian Lourido

**Affiliations:** 1 Whitehead Institute for Biomedical Research, Cambridge, Massachusetts, United States of America; 2 School of Chemistry and Biomedical Sciences Research Complex, University of St. Andrews and EaStCHEM, North Haugh, St Andrews, Fife, Scotland, United Kingdom; University at Buffalo, United States of America

## Abstract

*Toxoplasma gondii* is a parasite of humans and animals, and a model for other apicomplexans including *Plasmodium spp.*, the causative agents of malaria. Despite many advances, manipulating the *T. gondii* genome remains labor intensive, and is often restricted to lab-adapted strains or lines carrying mutations that enable selection. Here, we use the RNA-guided Cas9 nuclease to efficiently generate knockouts without selection, and to introduce point mutations and epitope tags into the *T. gondii* genome. These methods will streamline the functional analysis of parasite genes and enable high-throughput engineering of their genomes.

## Introduction

Tools for genome engineering are fundamental for the functional dissection of genes, and have greatly advanced our understanding of parasites for which homology to model organisms is often limited. In the case of *T. gondii*, a proliferation of tools for genetic manipulation, coupled to its relative ease of culture, have made it a model system for the cell biology of related apicomplexan parasites, including *Plasmodium spp.*: the causative agents of malaria. The recent generation of strains deficient in non-homologous end joining (NHEJ) through knocking out *KU80* (Δ*KU80*) has facilitated genome engineering by suppressing the high frequency of random integration naturally found in *T. gondii* [1], [2]. Nonetheless, these manipulations still rely on complex DNA constructs for homologous recombination, frequently require screening many clones for the correct rearrangement, and depend on selectable markers to enrich for the manipulated population. Furthermore, genome engineering remains for the most part restricted to select, lab adapted strains or the above-mentioned Δ*KU80* parasite lines.

Here, we demonstrate efficient genome editing in *T. gondii* using the prokaryotic CRISPR/Cas9 system, which has been shown to facilitate RNA-guided, site-specific DNA cleavage in diverse organisms ranging from yeast to mammalian cells [3]–[5]. This prokaryotic immune system taken from *Streptococcus pyogenes* has been reduced to its essential components, which include the Cas9 nuclease and a chimeric RNA (chiRNA) that directs this nuclease to its target [4]. The chiRNA consists of a 20 nt guide (also known as protospacer) sequence followed by an 85 nt chimeric sequence derived from the crRNA and tracrRNAs found in the bacterial system [4]. The 20 nt guide is perfectly complementary to the target sequence and must be followed in the target sequence by the nucleotides NGG, which are known as the protospacer-adjacent motif (PAM). This short motif allows the system to distinguish between the target and chiRNA-coding DNA sequences. Given its size, the guide sequence can be easily programmed to target diverse genomic loci for cleavage by Cas9. Double-stranded breaks introduced by Cas9 are repaired by the cellular machinery through either homologous recombination or the non-homologous end-joining pathway (NHEJ). DNA sequences homologous to the Cas9-targeted locus can be concurrently introduced to generate desired mutations [3]–[5].

Using a single plasmid system to express both the guide RNA and Cas9 nuclease, we can efficiently disrupt targeted loci in *T. gondii* without the need for selection. We provide evidence linking the efficiency of this process to the NHEJ pathway for DNA repair. We also demonstrate high rates of genome editing that allow us to introduce point mutations and epitope tags into the *T. gondii* genome. Together these methods dramatically improve our ability to manipulate the *T. gondii* genome and open the possibility of genetic manipulation in diverse genetic backgrounds and with greater throughput than previously possible.

## Materials and Methods

### Growth of Host Cells and Parasite Strains

All *T. gondii* strains used in this work are derived from the type I RH line. The Δ*KU80*Δ*HXGPRT* strain, referred to for clarity in this study as Δ*KU80*, was generously provided by V. Carruthers (University of Michigan, USA) and generated as previously published [1], [2]. Strains were maintained in Human Foreskin Fibroblast (HFF) cells grown in Dulbecco's Modified Eagle's Medium (DMEM) supplemented with 10% heat-inactivated fetal bovine serum and 10 µg/mL gentamicin.

### Plasmid Construction

All primer sequences can be found in Table S1. Plasmid pX330 encoding N-terminally FLAG tagged *Cas9* with nuclear localization sequences, and the guide RNA under mammalian promoters, as previously published [3]–[5],was generously provided by Tim Wang. The TgTUB1 promoter was cloned into the KpnI and NcoI sites upstream of *Cas9* using primers P1 and P2. Constructs with protospacers against *SAG1* (pU6-SAG1) and *PKG* (pU6-PKG), as well as a universal plasmid encoding BsaI sites in place of a protospacer, were synthesized by IDT (Figure S1). These constructs were amplified using P3 and P4, digested with NcoI and XbaI, and cloned into the PciI and XbaI sites of the *Cas9*-encoding plasmid. The construct targeting the *CDPK3* locus was generated by annealing oligos P25 and P26, phosphorylating the duplex, and cloning it into the BsaI-digested universal plasmid. The universal plasmid has been deposited with Addgene (ID no. 52694).

Oligos used to facilitate homologous recombination in *PKG* and *CDPK3* were generated by heating complementary 90- or 119- nucleotide oligomers ordered from IDT to 99 degrees for 2 minutes in a heat block, then removing the block from the heating apparatus and allowing the DNA to cool to room temperature over the course of a few hours. The sequences for these oligomers are listed in Table S1 as P5 and P6 for the *PKG* oligo and P7 and p8 for the *CDPK3* oligo.

The plasmid used to generate the allelic replacements of *PKG* was made by amplifying the 5′ end of the locus with P17 and P18 from RH genomic DNA and the 3′ end of the gene with P19 and P20 from RH cDNA. The two fragments were spliced together with AvrII and cloned into the PacI/AscI sites of pLIC-YFP-HXGPRT (provided by V. Carruthers, University of Michigan, USA), thus removing the YFP and introducing the two fragments. The gatekeeper residue in this construct was changed to code for T^761^M using site-directed mutagenesis.

Control plasmid 1 used in viability experiments was constructed by PCR-amplifying the pyrimethamine resistance cassette from pDHFR-TS [6] using primers P23 and P24, and cloned into the NsiI and SbfI sites of the universal plasmid in place of Cas9. Control plasmid 2 is the universal plasmid. Sequences for both control plasmids are provided in File S1.

### Construction of PKG allelic replacement strains

The previously described CDPK1^M^ strain [4], [7] was transfected with the PKG allelic replacement plasmid carrying either the wild-type or T^761^M gatekeeper, and linearized with StuI to improve homologous recombination. Parasites were selected with 25 µg/ml mycophenolic acid and 50 µg/ml xanthine prior to subcloning. Allelic replacements were identified using primers P21 and P22 against genomic DNA from established clonal lines.

### Transfections and Plaque Assays

Filtered *T. gondii* were washed and resuspended in Cytomix (10 mM KPO_4_, 120 mM KCl, 5 mM MgCl_2_, 25 mM HEPES, 2 mM EDTA) at 2×10^8^ parasites/ml. 5×10^7^ parasites were combined with 100 µg of CRISPR/Cas9 plasmid supplemented with 2 mM ATP, 5 mM GSH, and 150 µM CaCl_2_, with or without 40 µg of double-stranded oligonucleotide, in a final volume of 400 µl. The CaCl_2_ concentration used here matches the original formulation of Cytomix [8] but was added at the time of transfection to prevent the buildup of phosphate precipitates during buffer storage. Parasites were electroporated in 4 mm gap cuvettes (BTX Harvard Apparatus model no. 640) in an Electro Square Porator (BTX Harvard Apparatus). 200 parasites were added to confluent HFF monolayers in six-well dishes. In some cases, as indicated in the text, 2,000 or 20,000 parasites were used instead. If indicated, 0.2 µM Compound 2 (C2) or vehicle (DMSO) was added to these dishes 24 hpi. All plates were allowed to incubate for a total of eight days before staining with crystal violet. C2 was synthesized by N. Westwood and F. Tran as previously described [4], [9], [10].

### Fluorescent Microscopy

1.25×10^6^ *T. gondii* transfected with pU6-SAG1 were added to confluent monolayers of HFF cells grown on cover slips in 24-well dishes. Cells were fixed 24 hpi on ice with methanol for 2 minutes, then stained using mouse-anti-SAG1 (mAb DG52), rabbit-anti-TgAct1 [11], mouse-anti-Ty (mAb BB2 [12]), or mouse-anti-Flag (Sigma, F1804). Alexa-594-conjugated goat-anti-rabbit (Life Technologies, A11037) and Alexa-488-conjugated goat-anti-mouse (Life Technologies, A11029) were used as secondary antibodies. Antibodies against *Toxoplasma* were kindly provided by L. D. Sibley (Washington University School of Medicine, USA). Coverslips were mounted in Prolong Gold containing DAPI (Invitrogen P-36931) and imaged using an Eclipse Ti microscope (Nikon).

### Sequence Analysis

*T. gondii* transfected with pU6-SAG1 or pU6-PKG plasmids were grown in HFFs for two days, then pelleted and resuspended in 1X PCR buffer (Sigma P2192-1VL) in PBS plus 100 µg Proteinase K (Sigma). DNA was obtained by heating the samples to 37°C for 1 h, 50°C for 2 h, and 95°C for 15 min. Regions of *SAG1* and *PKG* surrounding their protospacers were amplified using primers P9 and P10, or P11 and P12, respectively (Table S1). A second round of amplification was then performed using primers P13 and P14 for *SAG1* or P15 and P16 for *PKG* to ensure purity of the product. The resulting fragments were cloned into pCR-II-Blunt-Topo (Invitrogen 45-0245) per the manufacturer's instructions. Clones were sent to EtonBioscience Inc. for sequencing with the primer M13F-21. Alignments were made using SeqMan pro Version 11.0.0.

### Statistics

Loss of SAG1 staining, transfection efficiency, viability, and Ty-tagging data were arcsine transformed, then analyzed using two-tailed Student's *t*-tests. The total numbers of plaques obtained with pU6-PKG with and without T^761^Q were compared using a one-tailed *t*-test.

## Results

### CRISPR/Cas9-mediated gene disruption

**Figure 1:**
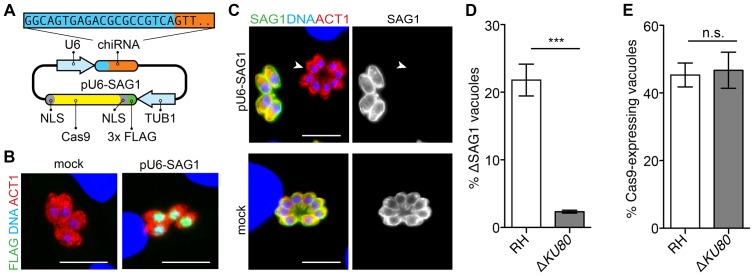
Targeted disruption of the *SAG1* locus using CRISPR/Cas9. (**A**) Strategy for constructing the CRISPR/Cas9 plasmid pU6-SAG1: chiRNA encoding a protospacer specific to *SAG1* (blue) and the Cas9 recognition motif (orange) was cloned under the upstream region of *T. gondii U6*. *CAS9* flanked by nuclear localization signals (NLS; gray) and a FLAG tag (green) was cloned under the control of the *TgTUB1* promoter. (**B**) Expression of Cas9 following transfection with or without pU6-SAG1. Parasites were fixed 24 hours post transfection and stained for parasite Actin (TgACT1; red), FLAG (green), and DAPI (DNA; blue). Scale bar is 10 µm. (**C**) Expression of SAG1 in wild-type RH parasites transfected with pU6-SAG1. Plasmid or mock-transfected parasites were allowed to grow for one lytic cycle before infecting monolayers for microscopy. Monolayers were fixed 24 hpi and stained for TgACT1 (red), SAG1 (green), and DNA. ΔSAG1 vacuole is indicated by an arrow. Scale bar is 10 µm. (**D**) Quantification of SAG1 knockout rates in RH and Δ*KU80*. Two-tailed Student's *t*-test; ***P<0.0005; means ± s.e.m., *n* = 3 experiments. (**E**) Quantification of transfection efficiency in the same populations 24 hours post transfection by monitoring Cas9 expression by immunofluorescence. Two-tailed Student's *t*-test; means ± s.e.m., *n* = 3 experiments.

To investigate whether the CRISPR/Cas9 system could be implemented in *T. gondii*, we adapted a single-plasmid system [4] to express chimeric RNA (chiRNA) and a FLAG-tagged Cas9 protein flanked by nuclear localization signals (NLS), both using parasite promoter sequences (Figure 1A). For Cas9 expression, we used the previously-characterized 5′UTR of the alpha-tubulin promoter [13]. Transcription of the chiRNA needs to be driven by an RNA polymerase III promoter to prevent polyadenylation. However, RNA pol III promoters have not been characterized in *T. gondii*. We identified the likely U6 gene through homology to the annotated *P. falciparum* gene (PlasmoDB.org), and cloned 500 bp upstream of the predicted transcription start site—corresponding to 980,755–981,251 bp on chromosome III of the GT1 genome—immediately upstream of the chiRNA sequence. The resulting plasmid was transfected into RH parasites. After 24 hours, immunofluorescent detection of the FLAG epitope revealed co-localization with DAPI staining of nuclear DNA, indicating the targeting of Cas9 to the parasite nucleus (Figure 1B).

As proof of principle, we targeted for disruption the 5′ end of the major tachyzoite surface protein (SAG1), which is dispensable for growth in fibroblasts and can be detected by a specific mAb [14]. To ensure target specificity, we compared the protospacer sequence to the *T. gondii* genome to identify homologous sequences that contained both the 8 bp seed sequence [15] and a PAM. Other than the intended target, the closest sequence to the designed protospacer contained at least 5 mismatches outside the seed sequence, and would not be targeted based on the reported specificity of CRISPR/Cas9 [4]. Wild-type RH parasites were transfected with the pU6-SAG1 construct and allowed to proceed through one infectious cycle before inoculating host cells adhered to coverslips for microscopy. Coverslips were stained 24 hpi for both SAG1 and parasite actin [11]. SAG1 expression was lost in a significant number of parasites within the population transfected with pU6-SAG1 (Figure 1C). In contrast, loss of SAG1 expression was never observed in mock-transfected parasites. Quantification of this observation revealed that transfection of pU6-SAG1 consistently yielded approximately 20% SAG1 knockout parasites in the absence of any selection (Figure 1D).

### Efficient gene disruption relies on NHEJ

We hypothesized that to prevent chromosomal instability within the haploid *T. gondii* genome, targeted double-stranded breaks generated by Cas9 must be repaired by non-homologous end joining (NHEJ). To test this hypothesis and further examine the mechanism behind CRISPR/Cas9-mediated gene disruption, we attempted to disrupt *SAG1* in Δ*KU80* parasites, which lack the KU80 DNA-binding protein required for NHEJ double-stranded break repair [1], [2]. Transfection of the pU6-SAG1 plasmid into Δ*KU80* parasites resulted in the detection of 10-fold fewer SAG1 deficient vacuoles compared to the wild-type RH strains (Figure 1D). To ensure transfection efficiencies were comparable between the two strains, Cas9 expression was monitored 24 hours post transfection and found to be equal (Figure 1E). These results suggest that efficient recovery of parasites following gene disruption requires a functional NHEJ pathway.

**Figure 2:**
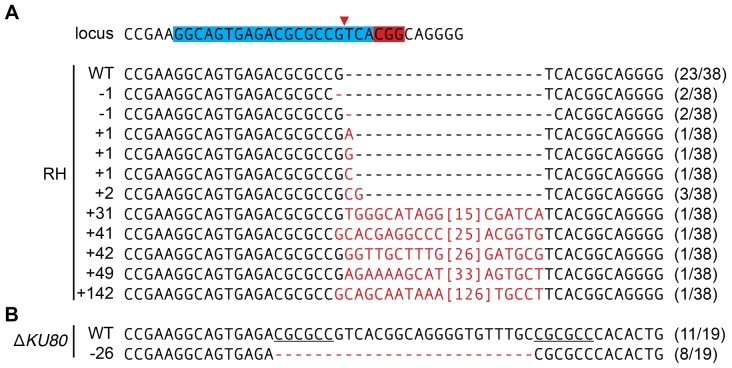
Analysis of SAG1 genomic locus following CRISPR/Cas9 disruption. DNA was isolated from parasites transfected with pU6-SAG1, and the region surrounding the protospacer was amplified by PCR, TOPO-cloned, and sequenced. The target locus is depicted with the protospacer and PAM highlighted (blue and red, respectively). The predicted cut site is indicated by a red arrow. The number of times a sequence was obtained out of the total number of sequences analyzed is shown in parentheses after each sequence.

To identify the types of mutations caused by CRISPR/Cas9, we amplified a portion of the SAG1 genomic locus using primers flanking the Cas9-targeted sequence. The amplified loci were cloned and individual clones were sequenced. Given the biases introduced by amplification and cloning, these results are not quantitative, but provide a clear picture of the types of mutations present in the population. Alignment of the sequences obtained from wild-type parasites transfected with pU6-SAG1 revealed an assortment of mutations precisely at the predicted cleavage site within the chiRNA-targeted region (Figure 2A). Although some of the identified mutations were similar to the small insertions and deletions caused by Cas9 targeting in mammalian systems [4], the five largest insertions were found to be perfectly homologous to portions of the pU6-SAG1 plasmid, consistent with an NHEJ-mediated repair of the double stranded break (Figure 2A).

In contrast to the diverse rearrangements found in the wild-type strain, only two types of sequences were recovered from the Δ*KU80* parasites transfected with pU6-SAG1: the endogenous locus and a 26 bp deletion (Figure 2B). Although we cannot be certain that the different instances represent independent events, repeated 6 bp sequences flanking the deletion (underlined; Figure 2B) suggest that the same rearrangement could have occurred various times through homologous recombination.

**Figure 3:**
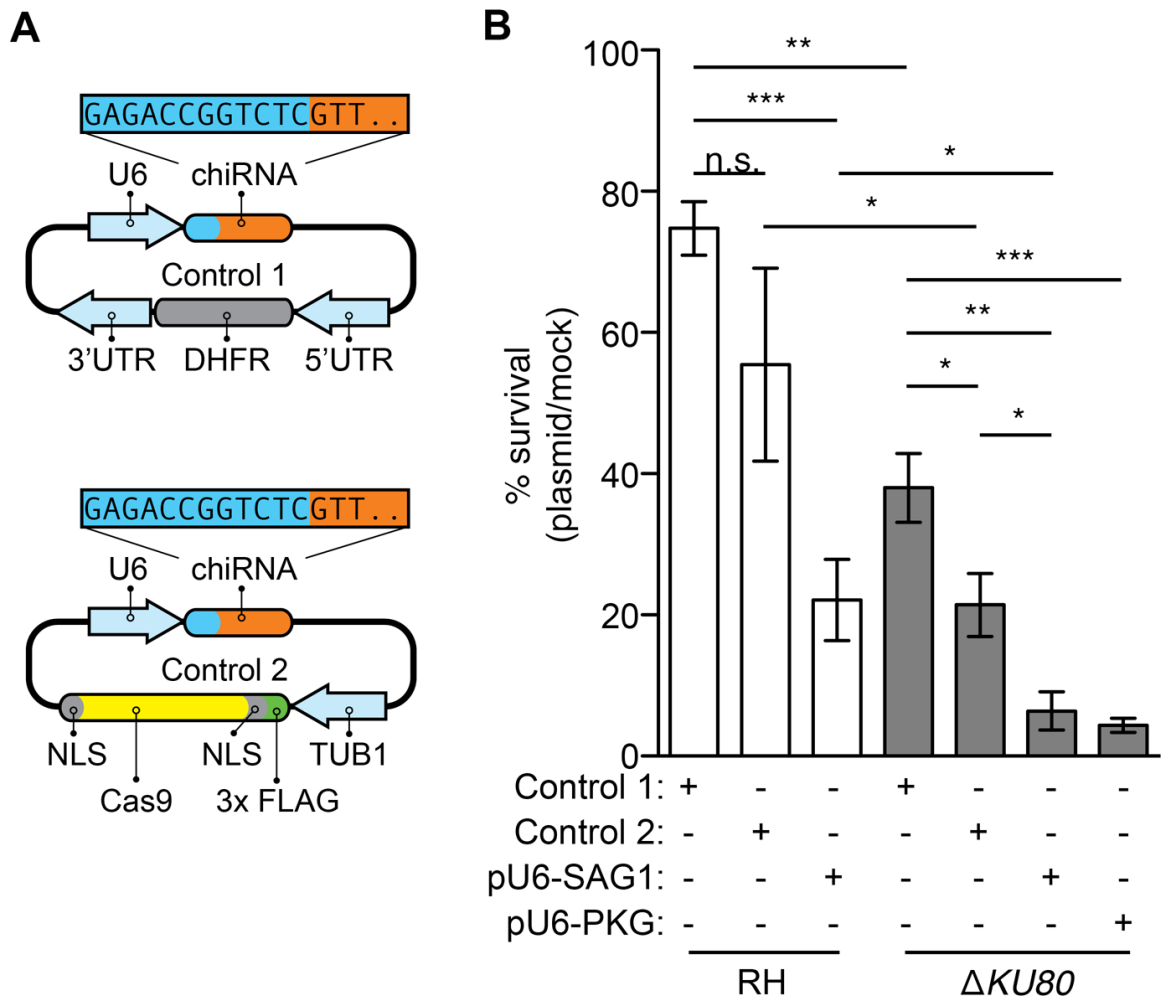
Parasite viability following CRISPR/Cas9. (**A**) Schematic representation of the control plasmids used to assess the effects of CRISPR/Cas9 on parasite viability. In Control 1, the Cas9 nuclease has been replaced with a pyrimethamine-resistance cassette containing a resistant allele of DHFR (gray) under the regulation of its endogenous 5′ and 3′ UTRs (blue). Control 2 is the universal plasmid lacking a specific targeting sequence. (**B**) Parasites were transfected with the control plasmids, pU6-SAG1 or pU6-PKG and immediately used to infect monolayers. The number of plaques generated after each transfection was compared to the plaquing efficiency of mock-transfected parasites to calculate percent viability. Student's *t*-test; ***P<0.0001;**P<0.005; *P<0.05; means ± s.e.m., *n* = 3 or 4 experiments.

We speculated that one potential cause for the observed decrease in gene disruption following transfection of Δ*KU80* parasites could be an inability to repair double stranded breaks. Consistent with this idea, these strains display increased sensitivity to DNA-damaging agents [1]. To test this hypothesis, we performed plaque assays to measure parasite viability immediately after transfection. We compared the number of plaques formed by mock-transfected parasites to those formed following transfection with pU6-SAG1 or either of two control plasmids. Both control plasmids were based on the pU6-SAG1 construct. The first plasmid lacks a protospacer specific to the *T. gondii* genome, and the Cas9 nuclease has been replaced with a pyrimethamine resistance cassette. The second plasmid only differs from the pU6-SAG1 plasmid in that it lacks the protospacer (Figure 3A). All DNA transfections decreased the viability of parasites, although this effect was smallest in wild-type parasites transfected with either of the control plasmids (Figure 3B). The Δ*KU80* strain was more susceptible than wild type to transfection with either the control plasmids or pU6-SAG1. Expression of the nuclease without a targeting protospacer (Control 2) had a marginally increased cost to viability that only achieved significance in the Δ*KU80* strain. In both strains, the greatest loss of viability was observed upon transfection with pU6-SAG1, which, as opposed to the controls, is expected to efficiently introduce double-stranded breaks at the *SAG1* locus. This suggests that loss of viability in response to CRISPR/Cas9 is multifactorial, including a modest effect from nuclease expression that is significantly exacerbated by the presence of a protospacer targeting the genome (pU6-SAG1). At present, we cannot explain the decrease in Δ*KU80* viability upon transfection with the control plasmids. However, consistent with its increased susceptibility to DNA damage, the Δ*KU80* strain may be unable to recover from CRISPR/Cas9-targeted double-stranded breaks in the absence of a template for homologous recombination, effectively resulting in a reduced frequency of observed gene disruption.

### Genome editing using CRISPR/Cas9

**Figure 4:**
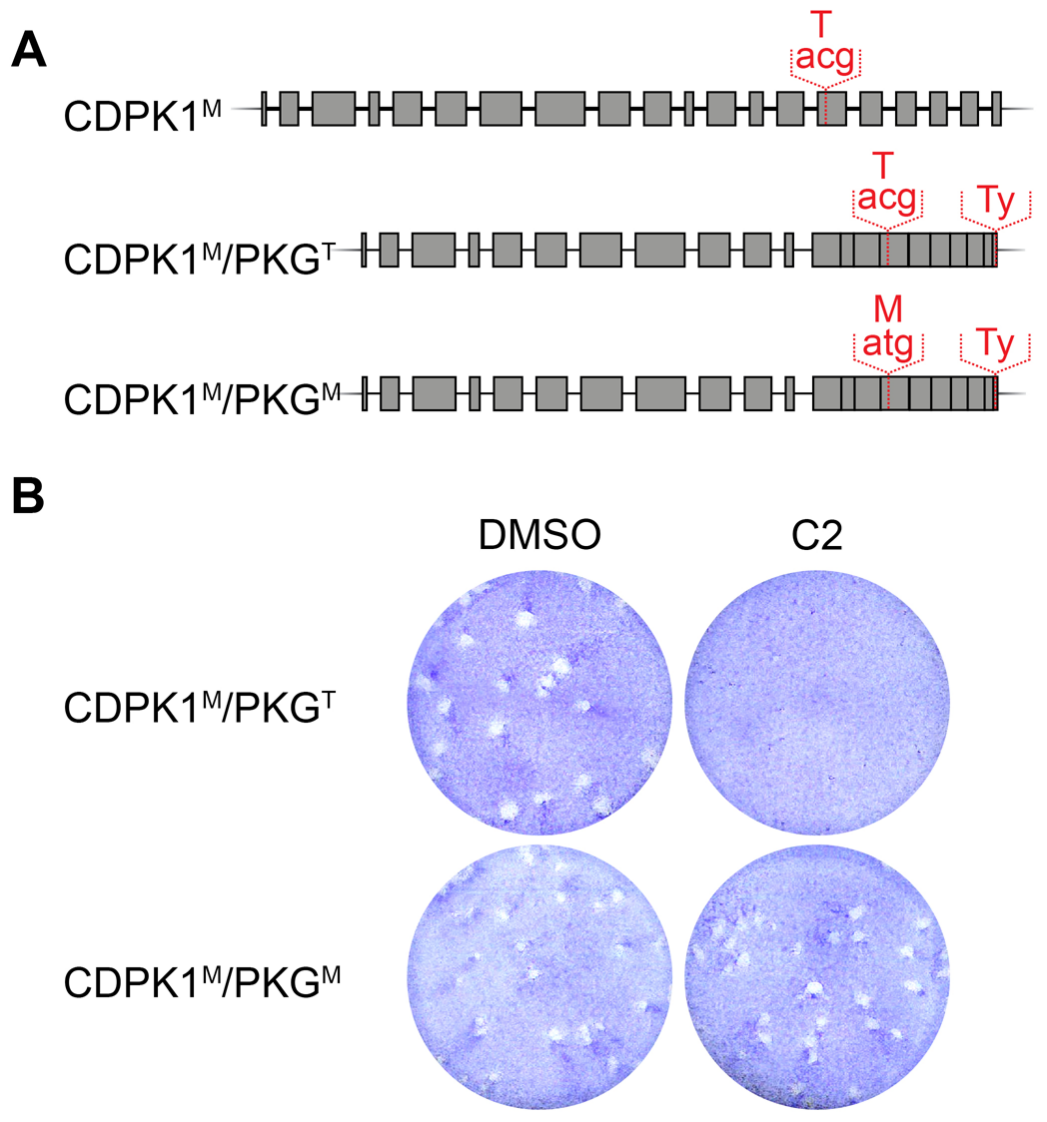
Generation of strains sensitive to C2 through the *PKG* locus. (**A**) Diagrams showing the wild-type *PKG* locus and strains with altered gatekeeper regions. The 3′ end of the endogenous *PKG* was replaced with the corresponding cDNA sequence harboring either the wild-type gatekeeper residue or the T^761^M mutation. (B) HFF monolayers were infected with either CDPK1^M^/PKG^T^ or CDPK1^M^/PKG^M^, and 0.2 µM C2 was added to the indicated wells 24 hours later. Plaques were allowed to develop for an additional 7 days before staining with crystal violet.

The expected reliance of the Δ*KU80* strain on homologous recombination to repair DNA breaks suggested it could be the ideal genetic background for direct genome editing using the CRISPR/Cas9 system. To quantitatively measure the rates of homologous recombination, we devised a system in which a single point mutation could confer antibiotic resistance. This system relies on the specificity of the trisubstituted pyrrole Compound 2 (C2), which has been shown to inhibit two kinases, CDPK1 and PKG, in *T. gondii* [9]. Inhibition by C2 relies on the expanded ATP-binding pocket resulting from the small gatekeeper residues present in these kinases. We isolated the effect of C2 on PKG by performing our analysis in a previously-generated Δ*KU80* strain where the endogenous *CDPK1* was replaced with an allele carrying a methionine gatekeeper [CDPK1M; 7]. As controls, we generated isogenic strains in the CDPK1^M^ background where the endogenous *PKG* was replaced with a Ty-tagged allele harboring either the wild-type gatekeeper or a T^761^M mutation (CDPK1^M^/PKG^T^ or CDPK1^M^/PKG^M^, respectively; Figure 4A). As expected, the bulky gatekeeper restored the ability of CDPK1^M^/PKG^M^ to grow and form plaques in the presence of C2 (Figure 4B).

**Figure 5:**
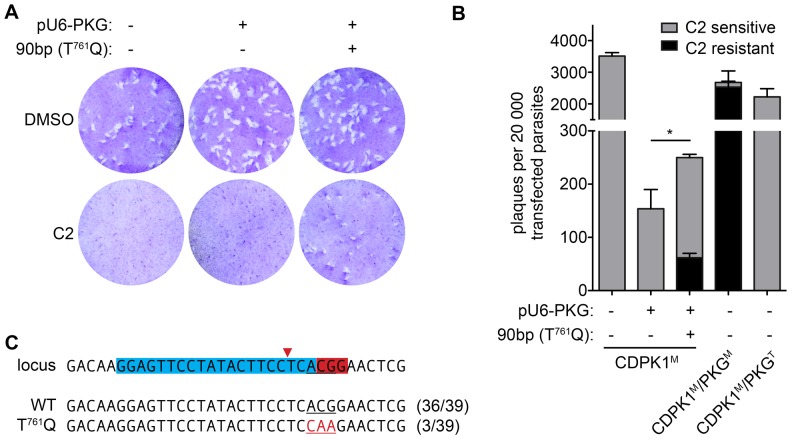
Genomic editing of the *PKG* locus using CRISPR/Cas9. (**A**) Plaque formation following transfection of CDPK1^M^ with or without pU6-PKG, with or without a 90 bp oligo centered on the T^761^Q mutation that confers resistance to C2. Transfected parasites were allowed to go through one infectious cycle prior to infection of plates with 200 parasites per well. 0.2 µM Compound 2 (C2) or vehicle control (DMSO) were added 24 hpi. Plaques were allowed to develop for an additional 7 days before staining with crystal violet. (**B**) Frequency of genome editing following CRISPR/Cas9. CDPK1^M^ parasites were transfected with the indicated construct in the presence or absence of the mutating oligo, and immediately plated. 0.2 µM C2, or vehicle control, were added 24 hpi, and plaques were allowed to form for 7 days before staining with crystal violet. Plaque numbers were compared to mock-transfected CDPK1^M^ or the isogenic strains carrying the wild-type or bulky-gatekeeper alleles of PKG. One-tailed Student's *t*-test; *P<0.05; means ± s.e.m., *n* = 3 experiments. (**C**) Analysis of the *PKG* locus following transfection with pU6-PKG and the recombination-facilitating oligo. The target locus is depicted with the protospacer and PAM highlighted (blue and red, respectively). The predicted cut site is indicated by a red arrow. A T^761^Q mutation was observed in 3 out of 39 sequences, and the remaining 36 sequences were wild type.

To target homologous recombination to the endogenous *PKG* allele, we designed a protospacer that would direct Cas9 to cut two bases upstream of the gatekeeper codon. As previously, the protospacer design was checked for specificity and found to be at least 5 bp different, outside the seed and PAM motifs, from all other sequences in the genome. We constructed the plasmid pU6-PKG, which is analogous to pU6-SAG1, containing the PKG-targeting chiRNA under the *U6* promoter and *Cas9* under the *TUB1* promoter. Transfection of pU6-PKG into the Δ*KU80* strain had the same effect on viability as the pU6-SAG1 plasmid (Figure 3), suggesting that the effect is independent of the locus targeted. To determine whether CRISPR/Cas9 could drive homologous recombination, we transfected CDPK1^M^ parasites with pU6-PKG in the presence or absence of a 90 bp DNA oligo centered on the T^761^Q mutation. Following one lytic cycle, parasites were used in plaque assays in the presence or absence of C2. No plaques were observed in the presence of C2 for mock transfections or parasites treated with the plasmid alone (Figure 5A). In contrast, parasites that received both the plasmid and the oligo yielded a significant number of plaques in the presence of C2, suggesting remarkable rates of homologous recombination (Figure 5A). To quantify this effect, parasites were placed into plaque assays immediately after transfection, and C2 was added to the wells indicated 24 hours later. Although addition of pU6-PKG resulted in the characteristic loss of viability, inclusion of the oligo moderately increased viability by the same proportion as the number of resulting C2 resistant plaques (Figure 5B). These results demonstrate that, in the absence of selection, CRISPR/Cas9 can initiate homologous recombination in up to a third of the resulting parasite population.

To confirm that C2 resistance was mediated by the intended mutation in PKG, the targeted locus was again amplified, TOPO cloned and sequenced. This analysis exclusively revealed either the wild-type locus or the expected T^761^Q mutation. Taken together, these results demonstrate that high rates of directed genome editing are possible in *T. gondii* using the CRISPR/Cas9 system.

### Tagging of endogenous loci using CRISPR/Cas9

**Figure 6:**
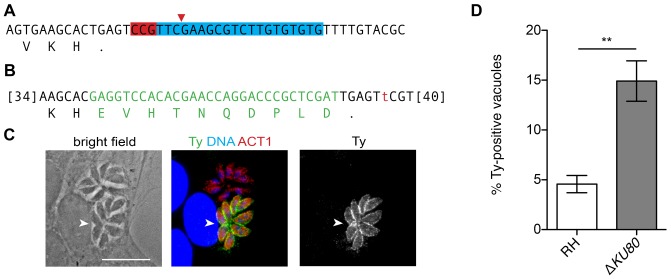
Epitope tagging of the endogenous *CDPK3* locus using CRISPR/Cas9. (**A**) Target 3′ end of the *CDPK3* locus with the protospacer and PAM highlighted (blue and red, respectively). (**B**) Homologous-recombination oligo used for tagging, indicating the Ty-tag sequence (green) and mutation of the PAM (red). Additional homology sequence is indicated in brackets. (**C**) Expression of Ty-tagged CDPK3 following transfection with pU6-CDPK3-Ct and the homologous-recombination oligo. Transfected parasites were allowed to grow for one lytic cycle before infecting monolayers for microscopy. Monolayers were fixed 24 hpi and stained for TgACT1 (red), Ty (green) and DNA. A Ty-expressing vacuole is indicated by an arrow. Scale bar is 10 µm. (**D**) Quantification of endogenous tagging rates in RH and Δ*KU80*. Two-tailed Student's *t*-test; **P<0.005; means ± s.e.m., *n* = 4 experiments.

Following our success introducing point mutations into the *T. gondii* genome, we attempted to epitope tag the *CDPK3* endogenous locus. Previous work has established that C-terminally tagged CDPK3 is functional and can complement the endogenous copy, suggesting there should be no detrimental consequences from similar manipulation of the endogenous locus [7], [16], [17]. We designed a protospacer that would target Cas9 to the 3′ end of the *CDPK3* locus, checking its specificity as described above (Figure 6A). We constructed plasmid pU6-CDPK3-Ct, analogous to the other targeting constructs and containing the CDPK3-targeting chiRNA under the *U6* promoter and *Cas9* under the *TUB1* promoter. To modify the locus, we synthesized a dsDNA oligo consisting of the sequence for the Ty epitope tag [12] followed by a stop codon and flanked by 40–43 bp of homology to introduce it in-frame with the *CDPK3* open reading frame (Figure 6B). Additionally, the oligo harbored a mutation in the PAM motif to ensure that following homologous recombination, the locus could no longer be targeted by the nuclease. The Δ*KU80* parasites were transfected with both pU6-CDPK3-Ct and the oligo and allowed to undergo a lytic cycle before inoculating host cells for microscopy. Samples were stained 24 hpi for both Ty and parasite actin. Vacuoles expressing Ty were readily observed in populations that had been transfected with both the oligo and the CRISPR/Cas9-targeting construct (Figure 6C), although they were never observed when either was omitted. Furthermore the pattern of Ty-staining matches the previously reported localization of CDPK3 at the periphery of the parasites [7], [16], [17].

To establish the rate of endogenous tagging in Δ*KU80* and wild-type parasites, we quantified the number of Ty-expressing vacuoles on the third day following transfection, as described above. The rate of endogenous tagging was approximately 5% in wild-type and 15% in Δ*KU80* parasites (Figure 6D), revealing an inverse trend to that of gene disruption (Figure 1D). These results are consistent with the NHEJ-mediated repair of CRISPR/Cas9 targeted double-stranded breaks in wild-type parasites, which are not expected to lead to in-frame incorporation of the Ty-tag.

## Discussion

Current methods for genome engineering of *T. gondii* rely on the generation of complex constructs for homologous recombination, which yield significant efficiency only in specific parasite strains and require antibiotic selection [1], [2], [18]. This has restricted this type of analysis to a relatively small number of parasite genes. Here, we present a genome engineering method based on the CRISPR/Cas9 system that permits, with a single plasmid, the rapid and efficient disruption of genomic loci. We demonstrate that Δ*KU80* parasite strains are significantly more vulnerable to these targeted disruptions than wild-type parasites, and we postulate that this vulnerability is due to the defect in this strain's ability to repair double-strand breaks using NHEJ. We further exploit the susceptibility of the Δ*KU80* strain as a genetic background in which to introduce targeted point mutations and epitope tags, and we show that genome editing can be achieved in 15–30% of the manipulated parasite population without the need for any form of selection. Together, these methods enhance our ability to manipulate the *T. gondii* genome and will enable high-throughput manipulation of a broad range of genetic loci.

Available tools for genome editing in *T. gondii* rely on antibiotic selection [1], [19] and are therefore difficult to compare to CRISPR/Cas9. However, our observations suggest that, beyond the obvious advantage of performing these manipulations in the absence of selection, CRISPR/Cas9 increases the rates of gene disruption by at least 100-fold over methods relying on homologous recombination [2]. We repeatedly observed knockout rates of approximately 20% when targeting CRISPR/Cas9 to the *SAG1* locus, which would make it possible to isolate knockouts directly from a transfected population. Not only will this method greatly expedite genetic manipulations, but it will also enable the generation of knockouts with reduced fitness, which would be lost in the course of selection using traditional methods. The single plasmid system used here can be easily reprogrammed to target virtually any genetic locus where a unique 20 bp sequence followed by a PAM can be identified, although ideal sequences should differ at two or more positions from other genomic loci [4], [20]. We have also generated a universal plasmid (Addgene ID no. 52694) that allows direct ligation of semi-complementary DNA oligos, thus streamlining the construction of the targeting constructs (Figure S1).

We compared the viability of wild-type and Δ*KU80* parasites transfected with CRISPR/Cas9 plasmids or with control plasmids lacking a targeting protospacer in the presence or absence of the Cas9 nuclease. We observed decreased viability in response to transfection of the SAG1-targetting plasmid (pU6-SAG1), although the loss was more pronounced in Δ*KU80* parasites, which exhibited a 96% decrease, compared to the 78% decrease found in wild-type parasites. In both strains, viability was improved in the absence of a targeting protospacer, and further improved in the absence of the nuclease. However, even in the absence of Cas9, the Δ*KU80* strain showed a significant decrease in viability, which we cannot attribute to the effects of CRISPR/Cas9. This effect may be caused by homology of the control plasmids to the genome or a more general response to foreign DNA in the Δ*KU80* strain, although the relatively low rates of homologous recombination in the absence of antibiotic selection [1] do not agree with the significant decrease in viability we report. The adverse effect of plasmid transfection and the predicted susceptibility of the Δ*KU80* strain to double-stranded breaks both contribute to the decrease in gene disruption frequency observed in the absence of NHEJ. It is not surprising that double-stranded DNA breaks are also detrimental to the wild-type strain, given that not all cells will be able to repair the lesions despite the efficiency of NHEJ. Following the initial crisis, parasites that underwent modification by CRISPR/Cas9 showed no secondary phenotypes in growth rate, plaque morphology, or replication that would suggest off-target effects of the CRISPR/Cas9 system. However, as with the severe bottlenecks that occur during antibiotic selection, genetic complementation will need to be performed to account for the presence of secondary mutations or polar effects.

The vulnerability of Δ*KU80* strains to targeted double-strand breaks suggested we could perhaps rescue them through homologous recombination and, in the process, introduce desired point mutations. We observed that a 90 bp oligonucleotide homologous to the targeted region of *PKG* indeed improved the survival rate of Δ*KU80* parasites transfected with pU6-PKG. Furthermore, a mutation included in the oligonucleotide was successfully incorporated into the parasite genome in a third of the surviving population. Similarly, targeting of the 3′ end of the *CDPK3* locus allowed us to incorporate an epitope tag into the open reading frame, which could be observed in 15% of the population three days post transfection, again in the absence of selection. As predicted the rate of endogenous tagging was significantly reduced in wild-type parasites, consistent with the NHEJ-mediated repair of targeted double-stranded breaks in this strain. These results highlight that although gene disruption is most readily achieved in wild-type strains, genome editing is most efficiently performed in the absence of NHEJ. However, deletion of *KU80* should be easily generated in any genetic background through CRISPR/Cas9. Similar manipulations should be feasible throughout the parasite genome with just minimal investment in the construction of the targeting plasmid.

The ease of CRISPR/Cas9 targeting, design and implementation, coupled with its efficiency, will greatly enhance our ability to manipulate the *T. gondii* genome. The methods described here should enable genetic manipulation of any transfection-competent strain and permit iterated modification of the parasite genome without concern for genetic markers or antibiotic selection. These tools have the potential to transform our analysis of the parasite genome by providing a truly multiplexable platform for genome manipulation.

## Supporting Information

Figure S1 **Diagram of CRISPR/Cas9 plasmid sequence.** (**A**) Sequence of the *SAG1* CRISPR insert synthesized by IDT. (**B**) Sequence of the *PKG* protospacer that replaces the *SAG1* protospacer to make pU6-PKG. (**C**) BsaI cloning sites that replace the *SAG1* protospacer to create a universal CRISPR plasmid.
(PDF)

Table S1 **Primers used in this study.**
(DOCX)

File S1 **Sequences of control plasmids.**
(PDF)
