# Supplementary material for: Efficient Genome Engineering of Toxoplasma gondii Using CRISPR/Cas9
Source: PLoS One. 2014 Jun 27;9(6):e100450. doi: 10.1371/journal.pone.0100450 (PMC4074098; doi:10.1371/journal.pone.0100450)
Supplement: Figure S1 — Diagram of CRISPR/Cas9 plasmid sequence. (A) Sequence of the SAG1 CRISPR insert synthesized by IDT. (B) Sequence of the PKG protospacer that replaces the SAG1 protospacer to make pU6-PKG. (C) BsaI cloning sites that replace the SAG1 protospacer to create a universal CRISPR plasmid. (PDF) [file pone.0100450.s001.pdf]

Figure S1

A

GCCCATGGGATGAGACAAAGTGCGCGAGTTGAAATCGTCGTGGGGACGATTTCACCGCGGCCACAT
NcoI

GTTGGAGACACTGAGGGCACACGGGAAACGCGAAAGATTTCAAATTAACGTACCCAAACGCGAAAG

CTTGCGCAGCATACACTCGAAGCGAACATCCCGAACCATCGAGAGGCGGAGAGCGATAAGTCTTTC

ACGCTGCGAAGTGTTGCGACGGCTGCGCCGCTGCACTGTGAATTGGGCGCCAATATTGCATCCTAG
U6 upstream region

GCCTGACGCGCCTCCTGCAGAACGCGAGACACTGGGATATGTAGAGCCAAGGGGGAAACCTTCGAA

CTCTCGAATGTCTTCTCTGACAAGAATCATATTTCCATCAGTTCTGTCAGATTTTCAAATGGCGAC

CTGCAGAGGCCTGCTTCCTCCCTGTGCGCTCTTCGAAGGGGCTTTCTGTCGCGCAGGGTCACCTCG

TCCCCGAAGGGGGTGTTTGCCTTCTGGTAAATGGGGATGTCAAGTTGGCAGTGAGACGCGCCGTCA
sag1 protospacer

GTTTTAGAGCTAGAAATAGCAAGTTAAAATAAGGCTAGTCCGTTATCAACTTGAAAAAGTGGCACC
Cas9 recognition sequence

GAGTCGGTGCTTTTTTTTTCTTTTTCTCTAGAGCATGCTAATGCG
PolIII terminator *XbaI* *priming sequence*

B

GGAGTTCCTATACTTCCTCA
pkg protospacer

C

GAGACCGGTCTC
BsaI cloning sites
