# Supplementary material for: Efficient Genome Engineering of Toxoplasma gondii Using CRISPR/Cas9
Source: PLoS One. 2014 Jun 27;9(6):e100450. doi: 10.1371/journal.pone.0100450 (PMC4074098; doi:10.1371/journal.pone.0100450)
Supplement: Table S1 — Primers used in this study. (DOCX) [file pone.0100450.s002.docx]

**Table S1. Primers used in this study.**

| **Primer** | **Sequence** |
| --- | --- |
| P1 | GCGGGTACCATGCATGTCCCGCGTTCGTG |
| P2 | GCGCCATGGTGTCGAAAAAGGGAATTCAAG |
| P3 | GCCCATGGGATGAGACAAAG |
| P4 | CGCATTAGCATGCTCTAGAG |
| P5 | GCAGTGAGAACGTTCCGCGACAAGGAGTTCCTATACTTCCTCCAAGAACTCGTCACAGGAGGCGAACTGTACGATGCCATCCGGAAGCTA |
| P6 | TAGCTTCCGGATGGCATCGTACAGTTCGCCTCCTGTGACGAGTTCTTGGAGGAAGTATAGGAACTCCTTGTCGCGGAACGTTCTCACTGC |
| P7 | GGAAATGATGCAGAAGATCTGCGACGTCAAAGTGAAGCACGAGGTCCACACGAACCAGGACCCGCTCGATTGAGTtCGTTCGAAGCGTCTTGTGTGTGTTTTGTACGCGCCACGTAACA |
| P8 | TGTTACGTGGCGCGTACAAAACACACACAAGACGCTTCGAACGaACTCAATCGAGCGGGTCCTGGTTCGTGTGGACCTCGTGCTTCACTTTGACGTCGCAGATCTTCTGCATCATTTCC |
| P9 | GTTTCGCTGCACCACTTC |
| P10 | GTTACAGCCTTTGATGTACAGC |
| P11 | CGGTTTCAATCGTGACTGAGTTCG |
| P12 | CATTCATGTGCAGACACTG |
| P13 | CTAGGTAATATGCACTGCAAG |
| P14 | CTGTGAGTTTGATGCCTGCC |
| P15 | GTTCTTGTCGCAGACGTGTG |
| P16 | CAAACCATCGACTAGTCCAC |
| P17 | GCGTTAATTAAGAGTTGTTTTGAAGAGACTTC |
| P18 | GAGGCCGTCCTAGGCTCATC |
| P19 | CTGTACGATGAGCCTAGGAC |
| P20 | TATGGCGCGCCTTAATCGAGCGGGTCCTGGTTCGTGTGGACCTCGAAATCCTTGTCCCAGTCATAC |
| P21 | AATGAGTCGATGCGTCTGGAG |
| P22 | CCAAGTGAGCAAGCATAGGC |
| P23 | CGATGCATCTAGCATGTCATTCGATTTTCACC |
| P24 | GCCCTGCAGGGCTCTAGAACTAGTGGATCG |
| P25 | AAGTTGCACACACAAGACGCTTCGAAG |
| P26 | AAAACTTCGAAGCGTCTTGTGTGTGCA |
