# Supplementary material for: Efficient Genome Engineering of Toxoplasma gondii Using CRISPR/Cas9
Source: PLoS One. 2014 Jun 27;9(6):e100450. doi: 10.1371/journal.pone.0100450 (PMC4074098; doi:10.1371/journal.pone.0100450)
Supplement: File S1 — Sequences of control plasmids. (PDF) [file pone.0100450.s003.pdf]

File S1. Sequences of Control Plasmids

> Control 1

ctagaggtacCATGCATctagcatgtcattcgattttcaccccccgcgtagttcctgtgtgtcattcgttgtcgagacaactct
gtcccgccccggtgctgttccatatgcgtgactttcccgcaatttttcagactttcaggaaagacaggctccggaacgatctcgtc
catgactggtaaatccacgacaccgcaatggcccccagcacctctatctctcgtgccaggggactaacgttgtatgcgtctgcgt
cttgtctttttgcattcgctttccaaaaaagagagccatccgttcccccgcacattcaacgccgcgagtgcggtttttgtctttttgag
tggtaggacgcttttcatgcgcgaactacgtggacattaagttccattctctttttcgacagcacgaaaccttgcattcaaacccgcc
cgcggaagatccgatcttgctgctgttcgcagtcccagtagcgtcctgtcggccgcgccgtctctGttggtgggcagccgctac
acctgttatCtgactgccgtgcgcgaaaatgacgccatttttgggaaaatcggggaacttcattctttaaaagtatgcggaggtttc
ctttttcttctgttcgtttctttttctcgggtttgataaccgtgttcgatgtaagcactttccgtctctcctccgtgctttgttcgacatcgag
accaggtgtgcagatccttcgcttgtcgatccggagacgcgtgtctcgtagaacctttttcattttaccacacggcagtgcggagca
ctgctctgagtgcagcagggacgggtgaagtttcgctttagtagtgcgtttctgctctacggggcgttgtcgtgtctgggaagAT
GCAGAAACCGGTGTGTCTGGTCGTCGCGATGACCCCCAAGAGGGGCATCGGC
ATCAACAACGGCCTCCCGTGGCCCCACTTGACCACAGATTTCAAACACTTTCG
TCGTGTGACAAAAACGACGCCCGAAGAAGCCAGTCGCCTGAACGGGTGGCTT
CCCAGGAAATTTGCAAAGACGGGCGACTCTGGACTTCCCTCTCCATCAGTCG
GCAAGAGATTCAACGCCGTTGTCATGGGACGGAAAAACTGGGAAAGCATGC
CTCGAAAGTTTAGACCCCTCGTGGACAGATTGAACATCGTCGTTTCCTCTTCC
CTCAAAGAAGAAGACATTGCGGCGGAGAAGCCTCAAGCTGAAGGCCAGCAG
CGCGTCCGAGTCTGTGCTTCACTCCCAGCAGCTCTCAGCCTTCTGGAGGAAGA
GTACAAGGATTCTGTCGACCAGATTTTTGTCGTGGGAGGAGCGGGACTGTAC
GAGGCAGCGCTGTCTCTGGGCGTTGCCTCTCACCTGTACATCACGCGTGTAGC
CCGCGAGTTTCCGTGCGACGTTTTCTTCCCTGCGTTCCCCGGAGATGACATTC
TTTCAAACAAATCAACTGCTGCGCAGGCTGCAGCTCCTGCCGAGTCTGTGTTC
GTTCCCTTTTGTCCGGAGCTCGGAAGAGAGAAGGACAATGAAGCGACGTATC
GACCCATCTTCATTTCCAAGACCTTCTCAGACAACGGGGTACCCTACGACTCC
GTGGTTCTCGAGAAGAGAAGGAAGACTGACGACGCAGCCACTGCGGAACCG
AGCAACGCAATGAGCTCCTTGACGTCCACGAGGGAGACAACTCCCGTGCACG
GGTTGCAGGCTCCTTCTTCGGCCGCAGCCATTGCCCCGGTGTTGGCGTGGATG
GACGAAGAAGACCGGAAAAAACGCGAGCAAAAGGAACTGATTCGGGCCGTT
CCGCATGTTCACTTTAGAGGCCATGAAGAATTCCAGTACCTTGATCTCATTGC
CGACATTATTAACAATGGAAGGACAATGGATGACCGAACGGGCGTTGGTGTC
ATCTCCAAATTCGGCTGCACTATGCGCTACTCGCTGGATCAGGCCTTTCCACT
TCTCACCACAAAGCGTGTGTTCTGGAAAGGGGTCCTCGAAGAGTTGCTGTGG
TTCATTCGCGGCGACACGAACGCAAACCATCTTTCTGAGAAGGGCGTGAAGA
TCTGGGACAAGAATGTGACACGCGAGTTCCTCGATTCGCGCAATCTCCCCCA
CCGAGAGGTCGGAGACATCGGCCCGGGCTACGGCTTCCAGTGGAGACACTTC
GGCGCGGCATACAAAGACATGCACACAGACTACACAGGGCAGGGCGTCGAC
CAGCTGAAGAATGTGATCCAGATGCTGAGAACGAATCCAACAGATCGTCGCA
TGCTCATGACTGCCTGGAATCCTGCAGCGCTGGACGAAATGGCGCTGCCGCC
TTGTCACTTGTTGTGCCAGTTCTACGTGAACGACCAGAAGGAGCTGTCGTGCA
TCATGTATCAGCGGTCGTGCGATGTCGGCCTCGGCGTCCCCTTCAACATCGCT
TCCTATTCGCTTTTGACGCTCATGGTTGCACACGTCTGCAACCTAAAACCTAA
GGAGTTCATTCACTTCATGGGGAACACGCATGTCTACACGAACCATGTCGAG
GCTTTAAAAGAGCAGCTGCGGAGAGAACCGAGACCGTTCCCCATTGTGAACA

TCCTCAACAAGGAACGCATCAAGGAAATCGACGATTTCACCGCCGAGGATTT
TGAGGTCGTGGGCTACGTCCCGCACGGACGAATCCAGATGGAGATGGCTGTC
TAGcggaaatacagaagctgcccgtctctcgttttcctctcttttcggagggatcagggagagtgcctcgggtcggagagagc
tgacgaggggggtgccagagacccctgtgtcctttatcgaagaaaagggatgactcttcatgtggcatttcacacagtctcacctcg
ccttgttttctttttgtcaatcagaacgaaagcgagttgcgggtgacgcagatgtgcgtgtatccactcgtgaatgcgttatcgttCt
gtatgccgctagagtgctggactgttgctgtctgcccacgacagcagacaactttccttctatgcaCttgcagGaTGAATT
CCTGCAGCCCGGGGGATCGATCCACTAGTTCTAGAGCcctgcaggggcgcctgatgcggta
ttttctccttacgcatctgtgcggtatttcacaccgcatacgtcaaagcaaccatagtacgcgccctgtagcggcgcattaagcgc
ggcgggtgtggtggttacgcgcagcgtgaccgctacacttgccagcgccctagcgcccgctcctttcgctttcttcccttcctttct
cgccacgttcgccggctttccccgtcaagctctaaatcgggggctccctttagggttccgatttagtgctttacggcacctcgaccc
caaaaaacttgatttgggtgatggttcacgtagtgggccatcgccctgatagacggtttttcgccctttgacgttggagtccacgttc
tttaatagtggactcttgttccaaactggaacaacactcaaccctatctcgggctattcttttgatttataagggattttgccgatttcgg
cctattggttaaaaaatgagctgatttaacaaaaatttaacgcgaattttaacaaaatattaacgtttacaattttatggtgcactctcag
tacaatctgctctgatgccgcatagttaagccagccccgacacccgccaacacccgctgacgcgccctgacgggcttgtctgct
cccggcatccgcttacagacaagctgtgaccgtctccgggagctgcatgtgtcagaggttttcaccgtcatcaccgaaacgcgc
gagacgaaagggcctcgtgatacgcctatttttataggttaatgtcatgataataatggtttcttagacgtcaggtggcacttttcgg
ggaaatgtgcgcggaacccctatttgtttatttttctaaatacattcaaatatgtatccgctcatgagacaataaccctgataaatgctt
caataatattgaaaaaggaagagtatgagtattcaacatttccgtgtcgcccttattcccttttttgcggcattttgccttcctgtttttgc
tcacccagaaacgctggtgaaagtaaaagatgctgaagatcagttgggtgcacgagtgggttacatcgaactggatctcaacag
cggtaagatccttgagagttttcgccccgaagaacgttttccaatgatgagcacttttaaagttctgctatgtggcgcggtattatcc
cgtattgacgccgggcaagagcaactcggtcgccgcatacactattctcagaatgacttggttgagtactcaccagtcacagaaa
agcatcttacggatggcatgacagtaagagaattatgcagtgctgccataaccatgagtgataacactgcggccaacttacttctg
acaacgatcggaggaccgaaggagctaaccgcttttttgcacaacatgggggatcatgtaactcgccttgatcgttgggaaccg
gagctgaatgaagccataccaaacgacgagcgtgacaccacgatgcctgtagcaatggcaacaacgttgcgcaaactattaac
tggcgaactacttactctagcttcccggcaacaattaatagactggatggaggcggataaagttgcaggaccacttctgcgctcg
gcccttccggctggctggtttattgctgataaatctggagccggtgagcgtggaagccgcggtatcattgcagcactggggcca
gatggtaagccctcccgtatcgtagttatctacacgacggggagtcaggcaactatggatgaacgaaatagacagatcgctgag
ataggtgcctcactgattaagcattggtaactgtcagaccaagtttactcatatatactttagattgatttaaaacttcatttttaatttaaa
aggatctaggtgaagatcctttttgataatctcatgaccaaaatcccttaacgtgagttttcgttccactgagcgtcagaccccgtag
aaaagatcaaaggatcttcttgagatcctttttttctgcgcgtaatctgctgcttgcaaacaaaaaaaccaccgctaccagcggtgg
tttgtttgccggatcaagagctaccaactctttttccgaaggtaactggcttcagcagagcgcagataccaaatactgtccttctagt
gtagccgtagttaggccaccacttcaagaactctgtagcaccgcctacatacctcgctctgctaatcctgttaccagtggctgctgc
cagtggcgataagtcgtgtcttaccgggttggactcaagacgatagttaccggataaggcgcagcggtcgggctgaacggggg
gttcgtgcacacagcccagcttggagcgaacgacctacaccgaactgagatacctacagcgtgagctatgagaaagcgccac
gcttcccgaagggagaaaggcggacaggtatccggtaagcggcagggtcggaacaggagagcgcacgagggagcttcca
gggggaaacgcctggtatctttatagtcctgtcgggtttcgccacctctgacttgagcgtcgatttttgtgatgctcgtcaggggg
cggagcctatggaaaaacgccagcaacgcggcctttttacggttcctggccttttgctggccttttgctcaCATGgGATGA
GACAAAGTGCGCGAGTTGAAATCGTCGTGGGGACGATTTCACCGCGGCCACA
TGTTGGAGACACTGAGGGCACACGGGAAACGCGAAAGATTTCAAATTAACGT
ACCCAAACGCGAAAGCTTGCGCAGCATACACTCGAAGCGAACATCCCGAACC
ATCGAGAGGCGGAGAGCGATAAGTCTTTCACGCTGCGAAGTGTTGCGACGGC
TGCGCCGCTGCACTGTGAATTGGGCGCCAATATTGCATCCTAGGCCTGACGC
GCCTCCTGCAGAACGCGAGACACTGGGATATGTAGAGCCAAGGGGGAAACC
TTCGAACTCTCGAATGTCTTCTCTGACAAGAATCATATTTCCATCAGTTCTGTC
AGATTTTCAAATGGCGACCTGCAGAGGCCTGCTTCCTCCCTGTGCGCTCTTCG
AAGGGGCTTTCTGTCGCGCAGGGTCACCTCGTCCCCGAAGGGGGTGTTTGCCT

TCTGGTAAATGGGGATGTCAAGTTGAGACCGGTCTCgttttagagctagaaatagcaagttaaa
ataaggctagtccgttatcaacttgaaaaagtggcaccgagtcggtgcTTTTTTTTTCTTTTTCt

> Control 2

ctagaggtacCATGCATGTCCCGCGTTCGTGaaattctctgcatcagcggagtgatcaggaatcatcgtctca
gcgggatgacgttgcggagcaggccggctcgcggtgggcagtcagatgccgaaggcgtaactcaggacggcttgcgctcat
cgcagaacaggggtggtgcctgcattgggtgcggttggtgatcctggttggaccggtggagatgcgcgcgcacgaaggggat
gtgtcagaaacattttgtttgttctctgtgaacttttagatgtgttaaaggcggcgaatattancagagagtcctccttgttggattctct
cttgaatttcgccctttccttctctttgcgagtctcgtagagaacaagcactcgttcgccgtccctgacgacgcaacccgcgcagaa
gacatccaccaaacggtgttacacaatcaccttgtgtgaagttcttgcggaaaactactcgttggcatttttttCTTGAATTCC
CTTTTTCGACACcATGGACTATAAGGACCACGACGGAGACTACAAGGATCAT
GATATTGATTACAAAGACGATGACGATAAGATGGCCCCAAAGAAGAAGCGG
AAGGTCGGTATCCACGGAGTCCCAGCAGCCGACAAGAAGTACAGCATCGGC
CTGGACATCGGCACCAACTCTGTGGGCTGGGCCGTGATCACCGACGAGTACA
AGGTGCCCAGCAAGAAATTCAAGGTGCTGGGCAACACCGACCGGCACAGCA
TCAAGAAGAACCTGATCGGAGCCCTGCTGTTCGACAGCGGCGAAACAGCCGA
GGCCACCCGGCTGAAGAGAACCGCCAGAAGAAGATACACCAGACGGAAGAA
CCGGATCTGCTATCTGCAAGAGATCTTCAGCAACGAGATGGCCAAGGTGGAC
GACAGCTTCTTCCACAGACTGGAAGAGTCCTTCCTGGTGGAAGAGGATAAGA
AGCACGAGCGGCACCCCATCTTCGGCAACATCGTGGACGAGGTGGCCTACCA
CGAGAAGTACCCCACCATCTACCACCTGAGAAAGAAACTGGTGGACAGCACC
GACAAGGCCGACCTGCGGCTGATCTATCTGGCCCTGGCCCACATGATCAAGT
TCCGGGGCCACTTCCTGATCGAGGGCGACCTGAACCCCGACAACAGCGACGT
GGACAAGCTGTTCATCCAGCTGGTGCAGACCTACAACCAGCTGTTCGAGGAA
AACCCCATCAACGCCAGCGGCGTGGACGCCAAGGCCATCCTGTCTGCCAGAC
TGAGCAAGAGCAGACGGCTGGAAAATCTGATCGCCCAGCTGCCCGGCGAGA
AGAAGAATGGCCTGTTCGGAAACCTGATTGCCCTGAGCCTGGGCCTGACCCC
CAACTTCAAGAGCAACTTCGACCTGGCCGAGGATGCCAAACTGCAGCTGAGC
AAGGACACCTACGACGACGACCTGGACAACCTGCTGGCCCAGATCGGCGACC
AGTACGCCGACCTGTTTCTGGCCGCCAAGAACCTGTCCGACGCCATCCTGCTG
AGCGACATCCTGAGAGTGAACACCGAGATCACCAAGGCCCCCCTGAGCGCCT
CTATGATCAAGAGATACGACGAGCACCACCAGGACCTGACCCTGCTGAAAGC
TCTCGTGCGGCAGCAGCTGCCTGAGAAGTACAAAGAGATTTTCTTCGACCAG
AGCAAGAACGGCTACGCCGGCTACATTGACGGCGGAGCCAGCCAGGAAGAG
TTCTACAAGTTCATCAAGCCCATCCTGGAAAAGATGGACGGCACCGAGGAAC
TGCTCGTGAAGCTGAACAGAGAGGACCTGCTGCGGAAGCAGCGGACCTTCGA
CAACGGCAGCATCCCCCACCAGATCCACCTGGGAGAGCTGCACGCCATTCTG
CGGCGGCAGGAAGATTTTTACCCATTCCTGAAGGACAACCGGGAAAAGATCG
AGAAGATCCTGACCTTCCGCATCCCCTACTACGTGGGCCCTCTGGCCAGGGG
AAACAGCAGATTCGCCTGGATGACCAGAAAGAGCGAGGAAACCATCACCCC
CTGGAACTTCGAGGAAGTGGTGGACAAGGGCGCTTCCGCCCAGAGCTTCATC
GAGCGGATGACCAACTTCGATAAGAACCTGCCCAACGAGAAGGTGCTGCCCA
AGCACAGCCTGCTGTACGAGTACTTCACCGTGTATAACGAGCTGACCAAAGT
GAAATACGTGACCGAGGGAATGAGAAAGCCCGCCTTCCTGAGCGGCGAGCA
GAAAAAGGCCATCGTGGACCTGCTGTTCAAGACCAACCGGAAAGTGACCGTG
AAGCAGCTGAAAGAGGACTACTTCAAGAAAATCGAGTGCTTCGACTCCGTGG

AAATCTCCGGCGTGGAAGATCGGTTCAACGCCTCCCTGGGCACATACCACGA
TCTGCTGAAAATTATCAAGGACAAGGACTTCCTGGACAATGAGGAAAACGAG
GACATTCTGGAAGATATCGTGCTGACCCTGACACTGTTTGAGGACAGAGAGA
TGATCGAGGAACGGCTGAAAACCTATGCCCACCTGTTCGACGACAAAGTGAT
GAAGCAGCTGAAGCGGCGGAGATACACCGGCTGGGGCAGGCTGAGCCGGAA
GCTGATCAACGGCATCCGGGACAAGCAGTCCGGCAAGACAATCCTGGATTTC
CTGAAGTCCGACGGCTTCGCCAACAGAAACTTCATGCAGCTGATCCACGACG
ACAGCCTGACCTTTAAAGAGGACATCCAGAAAGCCCAGGTGTCCGGCCAGGG
CGATAGCCTGCACGAGCACATTGCCAATCTGGCCGGCAGCCCCGCCATTAAG
AAGGGCATCCTGCAGACAGTGAAGGTGGTGGACGAGCTCGTGAAAGTGATG
GGCCGGCACAAGCCCGAGAACATCGTGATCGAAATGGCCAGAGAGAACCAG
ACCACCCAGAAGGGACAGAAGAACAGCCGCGAGAGAATGAAGCGGATCGAA
GAGGGCATCAAAGAGCTGGGCAGCCAGATCCTGAAAGAACACCCCGTGGAA
AACACCCAGCTGCAGAACGAGAAGCTGTACCTGTACTACCTGCAGAATGGGC
GGGATATGTACGTGGACCAGGAACTGGACATCAACCGGCTGTCCGACTACGA
TGTGGACCATATCGTGCCTCAGAGCTTTCTGAAGGACGACTCCATCGACAAC
AAGGTGCTGACCAGAAGCGACAAGAACCGGGGCAAGAGCGACAACGTGCCC
TCCGAAGAGGTCGTGAAGAAGATGAAGAACTACTGGCGGCAGCTGCTGAAC
GCCAAGCTGATTACCCAGAGAAAGTTCGACAATCTGACCAAGGCCGAGAGA
GGCGGCCTGAGCGAACTGGATAAGGCCGGCTTCATCAAGAGACAGCTGGTGG
AAACCCGGCAGATCACAAAGCACGTGGCACAGATCCTGGACTCCCGGATGA
ACACTAAGTACGACGAGAATGACAAGCTGATCCGGGAAGTGAAAGTGATCA
CCCTGAAGTCCAAGCTGGTGTCCGATTTCCGGAAGGATTTCCAGTTTTACAAA
GTGCGCGAGATCAACAACTACCACCACGCCCACGACGCCTACCTGAACGCCG
TCGTGGGAACCGCCCTGATCAAAAAGTACCCTAAGCTGGAAAGCGAGTTCGT
GTACGGCGACTACAAGGTGTACGACGTGCGGAAGATGATCGCCAAGAGCGA
GCAGGAAATCGGCAAGGCTACCGCCAAGTACTTCTTCTACAGCAACATCATG
AACTTTTTCAAGACCGAGATTACCCTGGCCAACGGCGAGATCCGGAAGCGGC
CTCTGATCGAGACAAACGGCGAAACCGGGGAGATCGTGTGGGATAAGGGCC
GGGATTTTGCCACCGTGCGGAAAGTGCTGAGCATGCCCCAAGTGAATATCGT
GAAAAAGACCGAGGTGCAGACAGGCGGCTTCAGCAAAGAGTCTATCCTGCC
CAAGAGGAACAGCGATAAGCTGATCGCCAGAAAGAAGGACTGGGACCCTAA
GAAGTACGGCGGCTTCGACAGCCCCACCGTGGCCTATTCTGTGCTGGTGGTG
GCCAAAGTGGAAAAGGGCAAGTCCAAGAAACTGAAGAGTGTGAAAGAGCTG
CTGGGGATCACCATCATGGAAAGAAGCAGCTTCGAGAAGAATCCCATCGACT
TTCTGGAAGCCAAGGGCTACAAAGAAGTGAAAAAGGACCTGATCATCAAGCT
GCCTAAGTACTCCCTGTTCGAGCTGGAAAACGGCCGGAAGAGAATGCTGGCC
TCTGCCGGCGAACTGCAGAAGGGAAACGAACTGGCCCTGCCCTCCAAATATG
TGAACTTCCTGTACCTGGCCAGCCACTATGAGAAGCTGAAGGGCTCCCCCGA
GGATAATGAGCAGAAACAGCTGTTTGTGGAACAGCACAAGCACTACCTGGAC
GAGATCATCGAGCAGATCAGCGAGTTCTCCAAGAGAGTGATCCTGGCCGACG
CTAATCTGGACAAAGTGCTGTCCGCCTACAACAAGCACCGGGATAAGCCCAT
CAGAGAGCAGGCCGAGAATATCATCCACCTGTTTACCCTGACCAATCTGGGA
GCCCCTGCCGCCTTCAAGTACTTTGACACCACCATCGACCGGAAGAGGTACA
CCAGCACCAAAGAGGTGCTGGACGCCACCCTGATCCACCAGAGCATCACCGG
CCTGTACGAGACACGGATCGACCTGTCTCAGCTGGGAGGCGACAAAAGGCCG

GCGGCCACGAAAAAGGCCGGCCAGGCAAAAAAGAAAAAGtaagaattcCTAGAG
CTCGCTGATCAGCCTCGACTGTGCCTTCTAGTTGCCAGCCATCTGTTGTTTGCC
CCTCCCCCGTGCCTTCCTTGACCCTGGAAGGTGCCACTCCCACTGTCCTTTCCT
AATAAAATGAGGAAATTGCATCGCATTGTCTGAGTAGGTGTCATTCTATTCTG
GGGGGTGGGGTGGGGCAGGACAGCAAGGGGGAGGATTGGGAAGAgAATAGC
AGGCATGCTGGGGAgcggccgcaggaacccctagtgatggagttggccactccctctctgcgcgctcgctcgctc
actgaggccgggcgaccaaaggtcgcccgacgcccgggctttgcccgggcggcctcagtgagcgagcgagcgcgcagctg
cctgcaggggcgcctgatgcggtattttctccttacgcatctgtgcggtatttcacaccgcatacgtcaaagcaaccatagtacgc
gccctgtagcggcgcattaagcgcggcgggtgtggtggttacgcgcagcgtgaccgctacacttgccagcgccctagcgccc
gctcctttcgctttcttcccttcctttctcgccacgttcgccggctttccccgtcaagctctaaatcgggggctccctttagggttccga
tttagtgctttacggcacctcgaccccaaaaaacttgatttgggtgatggttcacgtagtgggccatcgccctgatagacggtttttc
gccctttgacgttggagtccacgttctttaatagtggactcttgttccaaactggaacaacactcaaccctatctcgggctattcttttg
atttataagggattttgccgatttcggcctattggttaaaaaatgagctgatttaacaaaaatttaacgcgaattttaacaaaatattaa
cgtttacaattttatggtgcactctcagtacaatctgctctgatgccgcatagttaagccagccccgacacccgccaacacccgct
gacgcgccctgacgggcttgtctgctcccggcatccgcttacagacaagctgtgaccgtctccgggagctgcatgtgtcagagg
ttttcaccgtcatcaccgaaacgcgcgagacgaaagggcctcgtgatacgcctatttttataggttaatgtcatgataataatggttt
cttagacgtcaggtggcacttttcggggaaatgtgcgcggaacccctatttgtttatttttctaaatacattcaaatatgtatccgctca
tgagacaataaccctgataaatgcttcaataatattgaaaaaggaagagtatgagtattcaacatttccgtgtcgcccttattcccttt
ttgcggcattttgccttcctgtttttgctcacccagaaacgctggtgaaagtaaaagatgctgaagatcagttgggtgcacgagtgg
gttacatcgaactggatctcaacagcggtaagatccttgagagttttcgccccgaagaacgttttccaatgatgagcacttttaaagt
tctgctatgtggcgcggtattatcccgtattgacgccgggcaagagcaactcggtcgccgcatacactattctcagaatgacttgg
ttgagtactcaccagtcacagaaaagcatcttacggatggcatgacagtaagagaattatgcagtgctgccataaccatgagtgat
aacactgcggccaacttacttctgacaacgatcggaggaccgaaggagctaaccgcttttttgcacaacatgggggatcatgtaa
ctcgccttgatcgttgggaaccggagctgaatgaagccataccaaacgacgagcgtgacaccacgatgcctgtagcaatggca
acaacgttgcgcaaactattaactggcgaactacttactctagcttcccggcaacaattaatagactggatggaggcggataaagt
tgcaggaccacttctgcgctcggcccttccggctggctggtttattgctgataaatctggagccggtgagcgtggaagccgcggt
atcattgcagcactggggccagatggtaagccctcccgtatcgtagttatctacacgacggggagtcaggcaactatggatgaa
cgaaatagacagatcgctgagataggtgcctcactgattaagcattggtaactgtcagaccaagtttactcatatatactttagattg
atttaaaacttcatttttaatttaaaaggatctaggtgaagatcctttttgataatctcatgaccaaaatcccttaacgtgagttttcgttcc
actgagcgtcagaccccgtagaaaagatcaaaggatcttcttgagatcctttttttctgcgcgtaatctgctgcttgcaaacaaaaa
aaccaccgctaccagcggtggtttgtttgccggatcaagagctaccaactctttttccgaaggtaactggcttcagcagagcgca
gataccaaatactgtccttctagtgtagccgtagttaggccaccacttcaagaactctgtagcaccgcctacatacctcgctctgct
aatcctgttaccagtggctgctgccagtggcgataagtcgtgtcttaccgggttggactcaagacgatagttaccggataaggcg
cagcggtcgggctgaacggggggttcgtgcacacagcccagcttggagcgaacgacctacaccgaactgagatacctacag
cgtgagctatgagaaagcgccacgcttcccgaagggagaaaggcggacaggtatccggtaagcggcagggtcggaacagg
agagcgcacgagggagcttccagggggaaacgcctggtatctttatagtcctgtcgggtttcgccacctctgacttgagcgtcga
tttttgtgatgctcgtcaggggggcggagcctatggaaaaacgccagcaacgcggcctttttacggttcctggccttttgctggcct
tttgctcaCATGgGATGAGACAAAGTGCGCGAGTTGAAATCGTCGTGGGGACGATT
TCACCGCGGCCACATGTTGGAGACACTGAGGGCACACGGGAAACGCGAAAG
ATTTCAAATTAACGTACCCAAACGCGAAAGCTTGCGCAGCATACACTCGAAG
CGAACATCCCGAACCATCGAGAGGCGGAGAGCGATAAGTCTTTCACGCTGCG
AAGTGTTGCGACGGCTGCGCCGCTGCACTGTGAATTGGGCGCCAATATTGCA
TCCTAGGCCTGACGCGCCTCCTGCAGAACGCGAGACACTGGGATATGTAGAG
CCAAGGGGGAAACCTTCGAACTCTCGAATGTCTTCTCTGACAAGAATCATATT
TCCATCAGTTCTGTCAGATTTTCAAATGGCGACCTGCAGAGGCCTGCTTCCTC
CCTGTGCGCTCTTCGAAGGGGCTTTCTGTCGCGCAGGGTCACCTCGTCCCCGA

AGGGGGTGTTTGCCTTCTGGTAAATGGGGATGTCAAGTTGAGACCGGTCTCgtt
ttagagctagaaatagcaagttaaaataaggctagtccgttatcaacttgaaaaagtggcaccgagtcggtgcTTTTTTTT
TCTTTTTCt
